# Olfactory Dysfunction in Adults from Rio Grande do Norte: A Cross-Sectional Study

**DOI:** 10.1055/s-0043-1777448

**Published:** 2024-02-05

**Authors:** Henrique de Paula Bedaque, Emerson Kennedy Ribeiro de Andrade Filho, Caio de Oliveira Rabelo, José Eduardo Nóbrega Moura, Kelvin Leite Moura, José Diniz Junior, Maria Luisa Nobre Medeiros e Silva Guimarães

**Affiliations:** 1 Department of Surgery, Universidade Federal do Rio Grande do Norte, Natal, Rio Grande do Norte, Brazil; 2 Health Sciences Center, Universidade Federal do Rio Grande do Norte, Natal, Rio Grande do Norte, Brazil

**Keywords:** smell disorders, COVID-19, smell

## Abstract

**Introduction** Smell is one of the senses of the human body, and it can be affected by several factors, such as viral infections, traumatic brain injury, iatrogenesis, smoking, and neurodegenerative and systemic diseases.

**Objectives** The main goal of the present study is to describe the epidemiology of olfactory disorders in Rio Grande do Norte (RN). More specifically, to determine the prevalence of olfactory dysfunction and to identify the main risk factors related to these dysfunctions in the state's population.

**Methods** A total of 180 volunteers living in the RN underwent the Connecticut Clinical Research Center (CCCRC) smell test and a clinical and demographic questionnaire.

**Results** A total of 58.89% of the patients presented normosmia and 87.78% were classified as having between normal and mild hyposmia. A statistically significant relationship was found between worse performance in the test and nasal surgery ( *p* = 0.041) and the subjective feeling of not having an accurate sense of smell ( *p* = 0.006 on the right nostril). There was no statistical relationship between the olfactory status and the report of coronavirus disease 2019 (COVID-19) infection ( *p* = 0.254).

**Conclusion** The occurrence of altered sense of smell in our study was different from that reported in other studies that used the same test. The relationship with COVID-19 was not clear.

## Introduction

Olfaction is the chemical sensation of gaseous odorants colloquially referred to as the ability to smell. 1 It can be described as the stimulation of chemoreceptors located on the roof of the nasal cavity, which transduce chemical signals into electrical signals sent through the olfactory nerve, to the brain. 2 Thus, the sense of smell is of great importance in the relationship between human beings and the environment, helping to identify potentially dangerous foods and substances, as it makes it possible to create memories of danger and safety. 3

This function is also related to the perception of harmful environments, such as the presence of smoke, and to the chemical signalization of sexual selection. 4 The literature also points to a reciprocal relationship between olfactory function and depression, that is, individuals with olfactory dysfunction have more depressive symptoms than individuals with normal sense of smell, as well as individuals with severe depressive symptoms have worse sense of smell. 5

This very important sense can undergo changes, some of which can be quantified. A reduction in smell sensitivity is called hyposmia, as well as a complete loss of smell is called anosmia. 6

Several causes are related to changes in smell. Syed & Philpott 7 mention as possible causes: viral infection, chronic rhinosinusitis, traumatic brain injury (TBI), iatrogenesis, smoking, toxins, neurodegenerative diseases, tumors, congenital causes, and systemic diseases. In the current context, a specific viral infection to be evaluated is severe acute respiratory syndrome coronavirus 2 (SARS-CoV-2) (which causes coronavirus disease 2019 [COVID-19]), which has been associated with olfactory disturbances that persist after infection. 8

There are several tests to assess the olfactory function, which must be compatible with the dietary and cultural habits of the studied population. 9 It is also important that these tests assess smell quantitatively and qualitatively, that is, they must assess olfactory threshold and odor identification. 10 Thus, in 1988, the Connecticut Chemosensory Clinical Research Center (CCCRC) published a practical, accessible, and easy-to-apply test, in addition to being validated for Portuguese. 11 These features make the CCCRC test ideal for assessing the sense of smell in large populations.

The adaptation and validation of the CCCRC test in Brazil took place in 2020 11 and, due to this late validation, there is still a gap in the literature regarding the prevalence of hyposmia in the Brazilian population. Thus, the present study reflects the beginning of research aimed at understanding how olfactory disorders behave in Brazil.

The main goal of the study is to describe the epidemiology of olfactory disorders in Rio Grande do Norte (RN). More specifically, to determine the prevalence of olfactory dysfunction and to identify the main risk factors related to these dysfunctions in the state's population.

## Methods

This is an analytical, cross-sectional, and observational research with a quantitative approach. The research project was approved by the Research Ethics Committee of the Onofre Lopes University Hospital under CAAE 52106221.6.0000.5292 and followed the ethical aspects of research with humans contained in resolution 466/12 of the National Health Council. All participants signed the informed consent form.

The sample size was calculated based on the population of Rio Grande do Norte, over 18 years old, estimated by the Brazilian Institute of Geography and Statistics (IBGE) in 2020 (2,632,398 inhabitants). A margin of error of 5% and a confidence interval of 95% were assigned, resulting in a sample size of 180. The inclusion criteria were residents of Rio Grande do Norte over 18 years old who agreed to participate in the research. The exclusion criteria were current upper airway infection, temporary alteration of the cognitive pattern (as in uncontrolled psychiatric patients) or permanent (as in dementia syndromes) and terminally ill patients.

Volunteers were recruited at the Central Campus of the Federal University of Rio Grande do Norte (students, employees, and passers-by) and at the Onofre Lopes University Hospital (companions of patients from the largest tertiary hospital in the state).

The CCCRC smell test proposed by Cain et al. 10 was used, validated in Brazil by Fenólio et al. 11 and with the standardization suggested by Bedaque et al. 12 In addition, a demographic and clinical questionnaire (including the history of COVID-19 and time since infection) was applied. Data were collected between January and September 2022. Volunteers were classified according to test scores into: normosmia (6.00–7.00), mild hyposmia (5.00–5.75), moderate hyposmia (4.00–4.75), severe hyposmia (2.00–3.75), or anosmia (0.00–1.75).

Categorical variables were expressed as absolute (n) and relative (%) frequencies. For numerical variables, the median (M), 25th percentile (P25), 75th percentile (P75) and minimum and maximum values were calculated. To compare the smell test results, the non-parametric Mann-Whitney test for independent samples was applied. The choice of the test was based on the Shapiro-Wilk and Kolmogorov-Smirnov normality tests, which ruled out normal distribution in the evaluated groups. We considered it statistically significant when the *p* -value was less than 5%.

## Results

The CCCRC was applied to 180 volunteers. Participants were aged between 18 and 98 years, with a mean of 42.27 (± 15.38). The age and gender distribution of the study sample were compatible with the distributions of Rio Grande do Norte informed by the Instituto Brasileiro de Geografia e Estatística (IBGE) in 2020 ( Table 1 ).

**Table 1:** Demographic variables of study volunteers and Rio Grande do Norte

Variable	Sample		Rio Grande do Norte (IBGE)	
	n	%	n	%
Sex				
Female	97	53.90	1,369,438	52.02
Male	83	46.10	1,262,942	47.98
Age				
18–19 years old	1	0.56	110,785	4.21
20–29 years old	42	23.33	592,212	22.50
30–39 years old	41	22.78	596,648	22.67
40–49 years old	37	20.56	475,584	18.07
50–59 years old	32	17.78	395,294	15.02
60 years old or more	27	15	461,875	17.55

Abbreviation: IBGE, Instituto Brasileiro de Geografia e Estatística (Brazilian Institute of Geography and Statistics).

In each nostril, the result of the medians of the combined scores was within the normosmia range, as shown in Table 2 . Furthermore, no difference was observed between the medians for each nostril.

**Table 2:** Results of the Connecticut Chemosensory Clinical Research Center test in the population of Rio Grande do Norte

Evaluation	Median	Minimum	P25	P75	Maximum
Right side					
Olfactory threshold	6.0	1.0	5.0	7.0	7.0
Identification of odors	6.0	0.0	5.0	7.0	7.0
Final score	6.0	2.0	5.5	6.5	7.0
Left side					
Identification of odors	6.0	1.0	5.0	7.0	7.0
Identification of odors	6.0	1.0	5.0	7.0	7.0
Final score	6.0	2.0	5.5	6.5	7.0

The final classification of the volunteers resulted in normosmia in 58.89% of the right nostrils, 67.22% of the left nostrils, and 58.89% of the combined results ( Table 3 ). If we add to this value the volunteers who were classified as mild hyposmia, we reach 85.55% in the right nostrils, 87.78% in the left nostrils, and 87.78% in the final scores.

**Table 3:** Smell classification of volunteers submitted to the Connecticut Chemosensory Clinical Research Center test

Classification	Right side		Left side		Final score	
	n	%	n	%	n	%
Normosmia	106	58.89	121	67.22	106	58.89
Mild hyposmia	48	26.67	37	20.56	52	28.89
Moderate hyposmia	17	9.44	15	8.33	15	8.33
Severe hyposmia	9	5	7	3.89	7	3.89
Anosmia	0	0	0	0	0	0

The relationships between the clinical variables obtained in the questionnaire and the test result are shown in Table 4 . No statistically significant relationships were found regarding test performance in smoker volunteers, nasal disease, allergy, asthma, allergic rhinitis, history of TBI, recurrent epistaxis, heart disease, metabolic disease, and history of COVID-19. However, there was a statistically significant difference ( *p* = 0.041) between nasal surgery (in the right nostril and in the final score) and the subjective feeling of not feeling odors well (only in the right nostril, *p* = 0.006), with worse performance in these volunteers, we found a median of 6 for individuals who did not complain about smell and 5.5 for those who did.

**Table 4:** Relationship of different clinical variables with the result of the Connecticut Chemosensory Clinical Research Center test

Variable	Right side				Left side				Final score			
	Median	P25	P75	*P* -value	Median	P25	P75	*P* -value	Median	P25	P75	*P* -value
Smoking												
Yes ( *n* = 55)	6.0000	5.5000	6.5000	0.678	6.0000	5.5000	6.5000	0.238	5.7500	5.0000	6.5000	0.355
No ( *n* = 125)	6.0000	5.5000	6.5000		6.0000	5.5000	6.5000		6.0000	5.5000	6.2500	
Good sense of smell												
Yes ( *n* = 140)	6.0000	5.5000	6.5000	< 0.05	6.0000	5.5000	6.5000	0.825	6.0000	5.5000	6.5000	0.110
No ( *n* = 40)	5.5000	5.0000	6.0000		6.0000	5.5000	6.5000		5.8750	5.0000	6.2500	
Nasal disease												
Yes ( *n* = 30)	6.0000	5.5000	6.5000	0.969	6.0000	5.5000	6.5000	0.938	6.0000	5.6875	6.2500	0.912
No ( *n* = 150)	6.0000	5.0000	6.5000		6.0000	5.5000	6.5000		6.0000	5.4375	6.5000	
Nasal surgery												
Yes ( *n* = 12)	5.5000	4.6250	5.8750	< 0.05	5.5000	4.6250	5.8750	0.142	5.5000	5.0625	6.0000	< 0.05
No ( *n* = 168)	6.0000	5.5000	6.5000		6.0000	5.5000	6.5000		6.0000	5.5000	6.5000	
Allergies												
Yes ( *n* = 45)	6.0000	5.5000	6.500	0.952	6.0000	5.5000	6.5000	0.140	6.0000	5.3750	6.2500	0.280
No ( *n* = 135)	6.0000	5.5000	6.5000		6.0000	5.5000	6.5000		6.0000	5.5000	6.5000	
Asthma												
Yes ( *n* = 12)	6.0000	4.6250	6.5000	0.704	6.5000	5.5000	6.5000	0.963	5.8750	5.5000	6.5000	0.773
No ( *n* = 168)	6.0000	5.5000	6.5000		6.0000	5.5000	6.5000		6.0000	5.5000	6.2500	
Allergic rhinitis												
Yes ( *n* = 56)	6.0000	5.5000	6.5000	0.091	6.0000	5.5000	6.5000	0.284	6.0000	5.5000	6.5000	0.131
No ( *n* = 124)	6.0000	5.5000	6.5000		6.0000	5.5000	6.5000		6.0000	5.2500	6.2500	
History of traumatic brain injury												
Yes ( *n* = 6)	5.5000	4.0000	6.1250	0.205	5.7500	5.3750	6.2500	0.450	5.6250	4.6875	6.1875	0.263
No ( *n* = 174)	6.0000	5.5000	6.5000		6.0000	5.5000	6.5000		6.0000	5.5000	6.5000	
Recurrent epistaxis												
Yes ( *n* = 5)	6.0000	4.2500	6.7500	0.594	5.5000	4.0000	6.5000	0.325	6.0000	4.1250	6.5000	0.906
No ( *n* = 175)	6.0000	5.5000	6.5000		6.0000	5.5000	6.5000		6.0000	5.5000	6.5000	
Metabolic disease												
Yes ( *n* = 24)	6.0000	5.0000	6.5000	0.719	5.5000	4.5000	6.5000	0.159	6.0000	4.2500	6.5000	0.460
No ( *n* = 156)	6.0000	5.5000	6.5000		6.0000	5.5000	6.5000		6.0000	5.5000	6.2500	
History of COVID-19												
Yes ( *n* = 87)	6.0000	5.0000	6.5000	0.453	6.0000	6.0000	6.5000	0.128	6.0000	5.5000	6.2500	0.561
No ( *n* = 93)	6.0000	5.5000	6.5000		6.0000	5.5000	6.5000		6.0000	5.5000	6.5000	

**Fig. 1:**
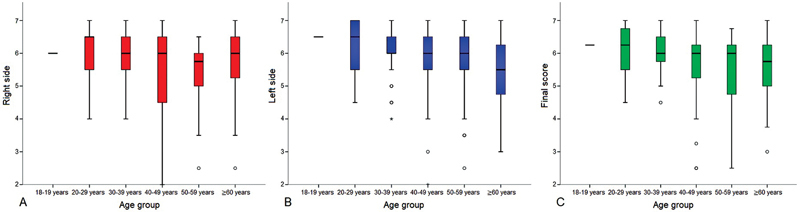
Distribution of the Connecticut Chemosensory Clinical Research Center test score according to age group on the right side (A), left side (B), and in the final score (C).

Elderly individuals (over 60 years old) had a final score lower than those under 60 years old ( Fig. 1 ). Furthermore, a weak negative correlation was found between final score and age (r = -0.215; *p* -value < 0.05).

In addition, no difference was found between the results of volunteers who had COVID-19 and those who did not ( Table 4 ). Pearson's Chi-square test demonstrated independence between categorical variables olfactory dysfunction (final score less than 6) and history of COVID-19 (χ ^2^ = 1.304; *v* = 1; *p* -value  = 0.254). Furthermore, the prevalence of olfactory dysfunction was higher in volunteers who did not have COVID-19 (45.16%) compared with those who had the disease (36.78%), and a weak negative correlation was found between the time (in months) that the patient had COVID-19 and the final score (r = -0.171; *p* = 0.113).

As for gender ( Table 5 ), a higher proportion of normal sense of smell was observed in females (62.89% in the final score; 73.20% on the left side; 63.92% on the right side) than in males (54.22% on the final score; 60.24% on the left side; 53.01% on the right side). In the left nostril, there was a better female performance ( *p* < 0.05), while in the final score, there was no difference between genders ( *p* = 0.068) ( Table 6 ).

**Table 5:** Distribution of classifications of volunteers in the Connecticut Chemosensory Clinical Research Center test by gender

Classification	Male						Female					
	Right side		Left side		Final score		Right side		Left side		Final score	
	n	%	n	%	n	%	n	%	n	%	n	%
Normosmia	44	53.01	50	60.24	45	54.22	62	63.92	71	73.20	61	62.89
Mild hyposmia	29	34.93	21	25.30	28	33.73	19	19.59	16	16.49	24	24.74
Moderate hyposmia	6	7.23	8	9.64	7	8.43	11	11.34	7	7.22	8	8.25
Severe hyposmia	4	4.82	4	4.82	3	3.61	5	5.15	3	3.09	4	4.12
Anosmia	0	0.00	0	0.00	0	0.00	0	0.00	0	0.00	0	0.00

**Table 6:** Relationship between sex and result in the Connecticut Chemosensory Clinical Research Center test

Sex	Right side				Right side				Final score			
	Median	P25	P75	*P* -value	Median	P25	P75	*P* -value	Median	P25	P75	*P* -value
Male ( *n* = 83)	6.00	5.00	6.00	0.474	6.00	5.50	6.50	< 0.05	6.00	5.50	6.50	0.068
Female ( *n* = 97)	6.00	5.50	6.50		6.00	5.00	7.00		6.00	5.50	6.50	

## Discussion

Smell disorders are very common in the general population and can lead to malnutrition, weight loss, food poisoning, depression, and other disorders. 13 During olfaction, the air flow takes volatile substances into the nasal cavities and, on the top of these, is the olfactory epithelium. 14 Olfactory disorders can be generated by: damage to this epithelium, which can occur due to different etiologies; or by conditions that affect the flow of air through the nasal cavity, preventing odorants from reaching the epithelium. 13 15

There are other smell tests available besides the CCCRC. Among them, we can mention the Sniffin' Sticks Test (SST), 16 which also evaluates olfactory threshold and identification of odors, but it has more odorants and requires odor-dispensing devices, which makes its application more difficult and less accessible. One can also cite the University of Pennsylvania Smell Identification Test (UPSIT), 17 which is one of the oldest validated smell tests in the world; however, despite being easy to apply, it only evaluates the identification of odors and not the olfactory threshold.

In addition to the smell tests, the P300 potential in the electroencephalogram (EEG) stands out. This potential is mainly related to cognitive functions and attention, but it is related to hearing, smell, eye movements and other functions, and may, in the future, be used to access olfactory function. 18 19 20 21

As for the test result, a higher relative frequency of altered smell (41.11%) was observed than other studies that also used the CCCRC. Toledano et al. 22 found a frequency of 11% in a Spanish sample ( *n* = 100), and Veyseller et al. 23 found a frequency of 18.4% in a Turkish sample ( *n* = 426). Even the CCCRC test validation study for Brazil 11 found a considerably lower relative frequency (17.4% on the right side and 16.5% on the left side) in a sample of 334 volunteers.

This different result may be related to several factors. Age can be highlighted, which is a factor related to worse performance in the test ,and its average was 42.27 years in our study, while it was 39.9 in the study by Fenólio et al. 11 and 36.7 in the study by Veyseller et al. 23 Other factors related to worse test results were more frequent in our sample than in Fenólio et al., 11 such as smoking (30.56% and 19.76%) and nasal surgery (6.67% and 2.99%).

However, in our sample, significant relationships were found regarding test performance in volunteers who underwent nasal surgery and who reported a subjective feeling of difficulty in smelling, a finding not observed in the study by Fenólio et al. 11 Our small sample, however compatible with the proposed sample size, may have limited the evaluation of certain relationships, as many of the risk factors for altered sense of smell cited in the literature were found in a very limited number of volunteers.

In addition, for questions regarding COVID-19, we were subject to memory bias, as well as patients who claimed not to have COVID-19 could have presented the disease in an oligosymptomatic way without its diagnosis.

Knowing that the literature has been indicating the presence of olfactory alterations as a complication of COVID-19, 8 our research tried to evaluate the effect of the long-term infection by COVID-19 by the CCCRC. However, we found no significant difference in test performance between volunteers who did and those that did not have the disease. This may be related to our data collection period, as the Omicron variant (the most frequent during data collection) does not cause changes in the sense of smell as frequently as the variants that circulated at the beginning of the pandemic. 24

## Conclusion

The CCCRC test adapted for Brazil proved to be easy to apply and low cost, so it can be easily reproduced by other large-scale study centers. In our sample of volunteers from Rio Grande do Norte, a prevalence of olfactory alterations of 41.11% was found. This value was higher than that found in other studies that used the same test. It was possible to relate olfactory dysfunction with advanced age, male gender, nasal surgery, and subjective impaired sense of smell. Furthermore, the relationship with SARS-CoV-2 infection was not clear.
